# Supplementary material for: N-Acetylcysteine Antagonizes NGF Activation of TrkA through Disulfide Bridge Interaction, an Effect Which May Contribute to Its Analgesic Activity
Source: Int J Mol Sci. 2023 Dec 22;25(1):206. doi: 10.3390/ijms25010206 (PMC10778962; doi:10.3390/ijms25010206)
Supplement: Supplementary file 1 [file ijms-25-00206-s001.zip › ijms-2756740-supplementary.pdf]

Supplementary File

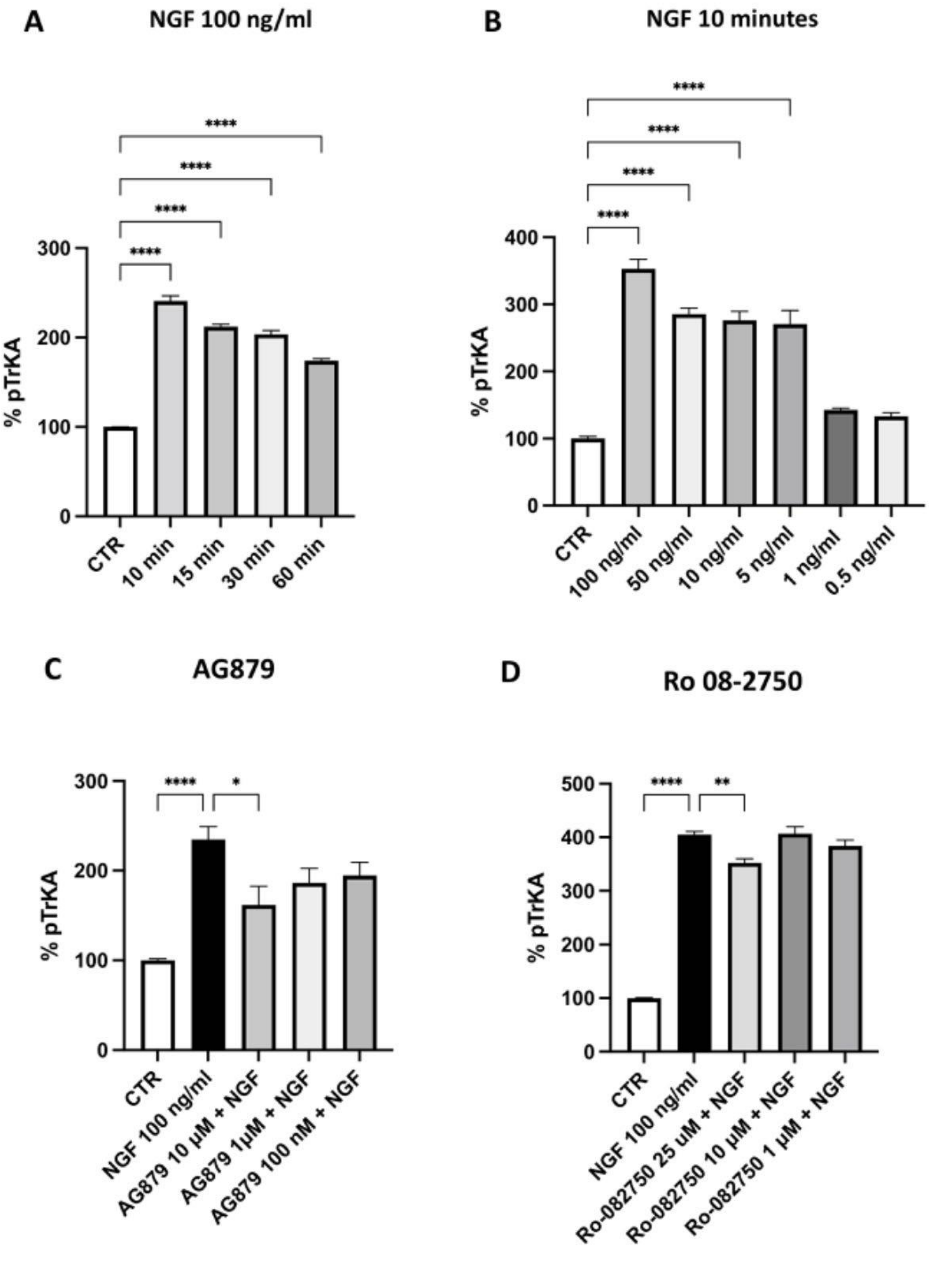

Figure S1. Time (A) and concentration (B) dependency of NGF for TrkA receptor activation in SH-SY5Y neuroblastoma cells and inhibition by two reference compounds (C and D). Each value represents the mean ± S.E.M. of independent experiments with respect to the control (100%). ****$p<0.0001$, **$p \leq 0.01$, *$p \leq 0.05$; Dunnett's Multiple Comparison post-hoc test (A) and Tukey's Multiple Comparison post-hoc test (B), n=3.
